# Bibliometric analysis of the interplay between epilepsy and microglia: trends, hotspots, and emerging research areas

**DOI:** 10.3389/fneur.2024.1439823

**Published:** 2024-10-09

**Authors:** Shouye Zhang, Xiaotong Yang, Yuping Wang

**Affiliations:** ^1^Department of Neurology, The First Hospital, Hebei Medical University, Shijiazhuang, China; ^2^Department of Neurology, Xuanwu Hospital, Capital Medical University, Beijing, China; ^3^Beijing Key Laboratory of Neuromodulation, Beijing, China; ^4^Collaborative Innovation Center for Brain Disorders, Capital Medical University, Beijing, China; ^5^Hebei Hospital of Xuanwu Hospital, Capital Medical University, Shijiazhuang, China; ^6^Neuromedical Technology Innovation Center of Hebei Province, Shijiazhuang, China

**Keywords:** bibliometric analysis, VOSviewer, epilepsy, microglia, neuroinflammation, research trends

## Abstract

**Background:**

Epilepsy, a common neurological disorder, has been increasingly associated with neuroinflammation, especially microglia activation. To gain insights into the research trends and patterns in this intersection, we conducted a bibliometric analysis of studies published between 2005 and 2024. Using the Web of Science Core Collection, we identified 1,229 relevant articles and reviews, focusing on the relationship between epilepsy and microglia.

**Methods:**

We employed the Bibliometrix R package and VOSviewer to analyze the data. Our search strategy combined epilepsy-related terms with microglia and microglial cell keywords. The analysis encompassed publication trends, country and institutional contributions, journal sources, authors, keywords, and thematic evolution.

**Results:**

The number of publications has steadily increased, particularly after 2019, indicating growing research interest. The United States, China, and Germany emerged as the most productive countries, with extensive collaboration between China and the United States. Epilepsia and Journal of Neuroinflammation were the most influential journals. Aronica E, Vezzani A, and Engel T were the most prolific authors. Thematic analysis revealed clusters focused on temporal lobe epilepsy, epilepsy-related disorders, and microglia activation. Over the past several years, research has shifted from fundamental brain function studies to *in-vivo* investigations of specific molecular mechanisms. The CSTB (−) mouse model and NF-κB signaling pathway both merit further in-depth investigation.

**Conclusion:**

In conclusion, this bibliometric study reveals a surge in epilepsy-microglia research, led by key countries, journals, and researchers. Temporal lobe epilepsy, epilepsy-related disorders, and microglia activation are focal themes. Future directions include exploring microglia activation mechanisms, utilizing animal models, and interdisciplinary approaches.

## 1 Introduction

The interplay between epilepsy and neuroinflammation, particularly the role of microglia, has garnered significant attention in recent years. Microglia, the resident immune cells of the central nervous system (CNS), play a crucial role in maintaining homeostasis (1). However, when inappropriately activated, microglia can release a wide range of inflammatory mediators that have the potential to disrupt neuronal signaling and contribute to the development and progression of neurological disorders (2). A study by Vezzani et al. (3) reported microglial activation and upregulation of inflammatory markers in the hippocampus and cortex of epileptic mice. Similarly, Megumi et al. (4) found increased microglial density and activation in the brains of patients with temporal lobe epilepsy (TLE).

Bibliometrics began as a relatively simple means of counting publications and citations to assess the productivity and impact of researchers and institutions (5). However, over the years, it has evolved into a multifaceted discipline that incorporates various statistical techniques, network analysis, and alternative metrics. This evolution has been driven largely by the digital revolution and the availability of large-scale repositories of scholarly data. The advent of big data and data mining techniques has further propelled the development of bibliometrics. By analyzing vast repositories of scholarly data, researchers can identify patterns, trends, and emerging research areas. This information is invaluable for strategic planning, resource allocation, and policy making (6–8). In this paper, we aim to employ bibliometric techniques to analyze the relationship between epilepsy and microglia. By mining large-scale databases of scholarly publications, we will identify the key research trends, hotspots, and emerging areas of investigation. We will also examine the collaboration patterns and trends in this field, aiming to provide insights into how research is conducted and supported.

## 2 Materials and methods

### 2.1 Data collection and retrieval strategy

**Figure 1:**
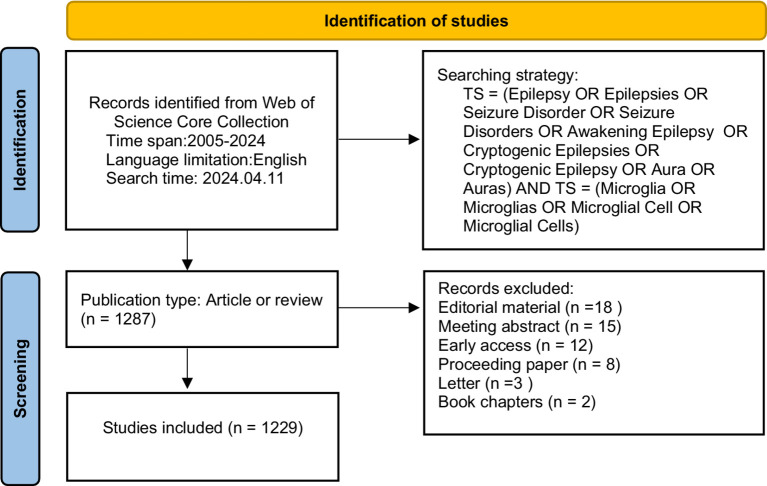
Flow diagram of Identification.

The Web of Science Core Collection (WOSCC) is widely acknowledged as a fundamental data source for bibliometric analysis and research evaluation. We collected relevant literature as of April 11, 2024, time span = 2005–2024, and applied restrictions to the English language from the WOS Core Collection. Two researchers (SYZ and XTY) conducted the literature search independently. Only articles (*n* = 985) and reviews (*n* = 244) were considered for inclusion. The search terms were provided in the following manner: TS = (“Epilepsy” OR “Epilepsies” OR “Seizure Disorder” OR “Seizure Disorders” OR “Awakening Epilepsy” OR “Cryptogenic Epilepsies” OR “Cryptogenic Epilepsy” OR “Aura” OR “Auras”) AND TS = (“Microglia” OR “Microglias” OR “Microglial Cell” OR “Microglial Cells”). Figure 1 shows the detailed retrieval strategy.

### 2.2 Statistical analysis

Bibliometrix (9) is an R package that performs bibliometric analyses, including visualizing data, analyzing networks, and evaluating research performance. It helps to identify important articles, authors, and trends in science. VOSviewer is a complementary tool that is specialized in the construction and visualization of bibliometric networks. It provides a clear overview of the research landscape of a field by creating maps based on co-occurrences or citation links. They are an invaluable resource for researchers and scholars across a wide range of fields.

The bibliometric analysis was carried out with the use of the bibliometric package (version 4.1.4), R (version 4.3.3), and VOSviewer (version 4.3.3). Using Microsoft Excel 2019, accumulated and annual numbers of articles published were plotted.

## 3 Results

### 3.1 Publication trends

**Figure 2:**
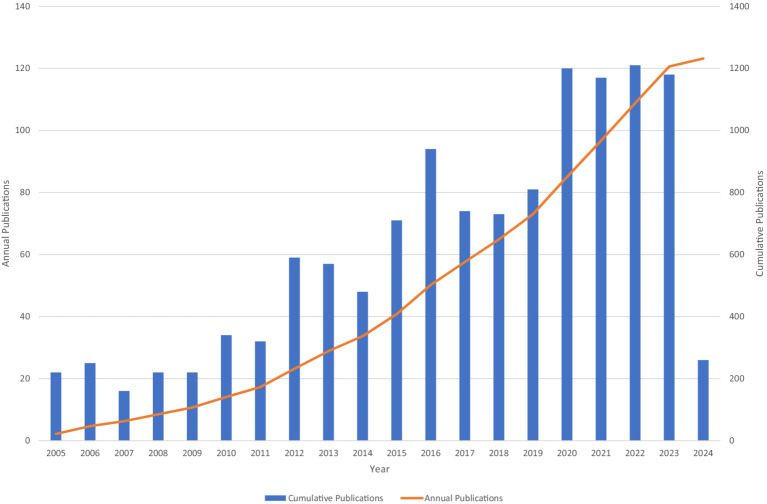
Cumulative and annual publications.

A total of 1,229 studies qualified for inclusion and were included between 2005 and 2024 in the analysis, comprising of 985 articles and 244 reviews. Overall, the number of publications tends to increase, although some years fluctuate slightly. This demonstrates that research interest has continued and grown in the correlation between microglia and epilepsy, specifically after 2019 (Figure 2).

### 3.2 Countries and affiliations

**Figure 3:**
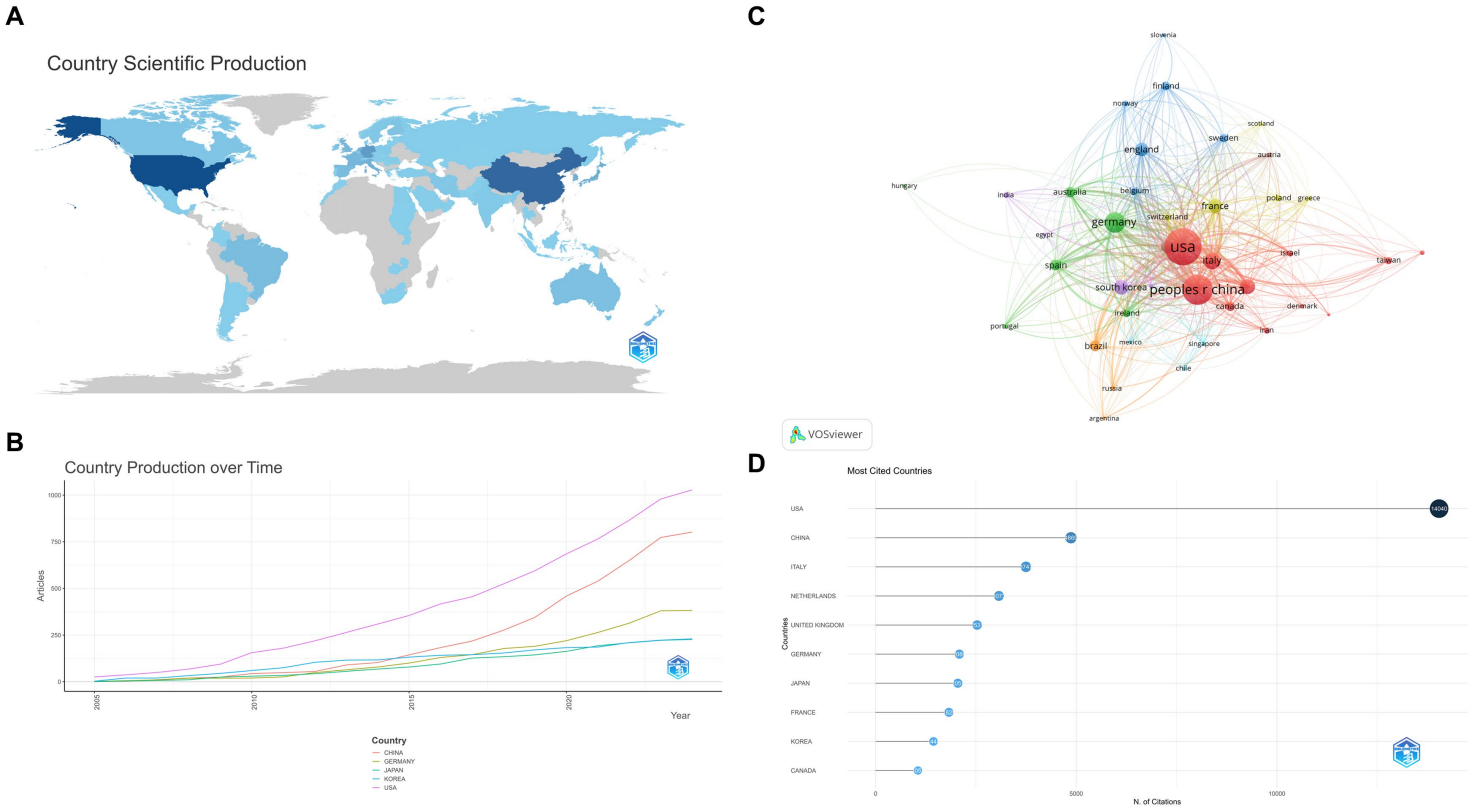
(A) World Contribution. Posts become darker in color as they increase. (B) Country Production over Time. (C) Citations of country. The nodes represent countries, with larger circles indicating a greater number of citations. (D) Citations of country. The darker the color and the larger the circle, the greater the number of citations.

There were 1,582 affiliations from 68 Countries participated in this field. All countries contributed as shown in Figure 3A. The most productive countries were the United States, China and Germany. Notably, the latter two have experienced significant production growth since 2015 (Figure 3B). In terms of citations, the top five cited countries: United States (14040), China (4869), Italy (3747), Netherlands (3073), and United Kingdom (2531). The United States remains well in the lead (Figures 3C,D).

**Figure 4:**
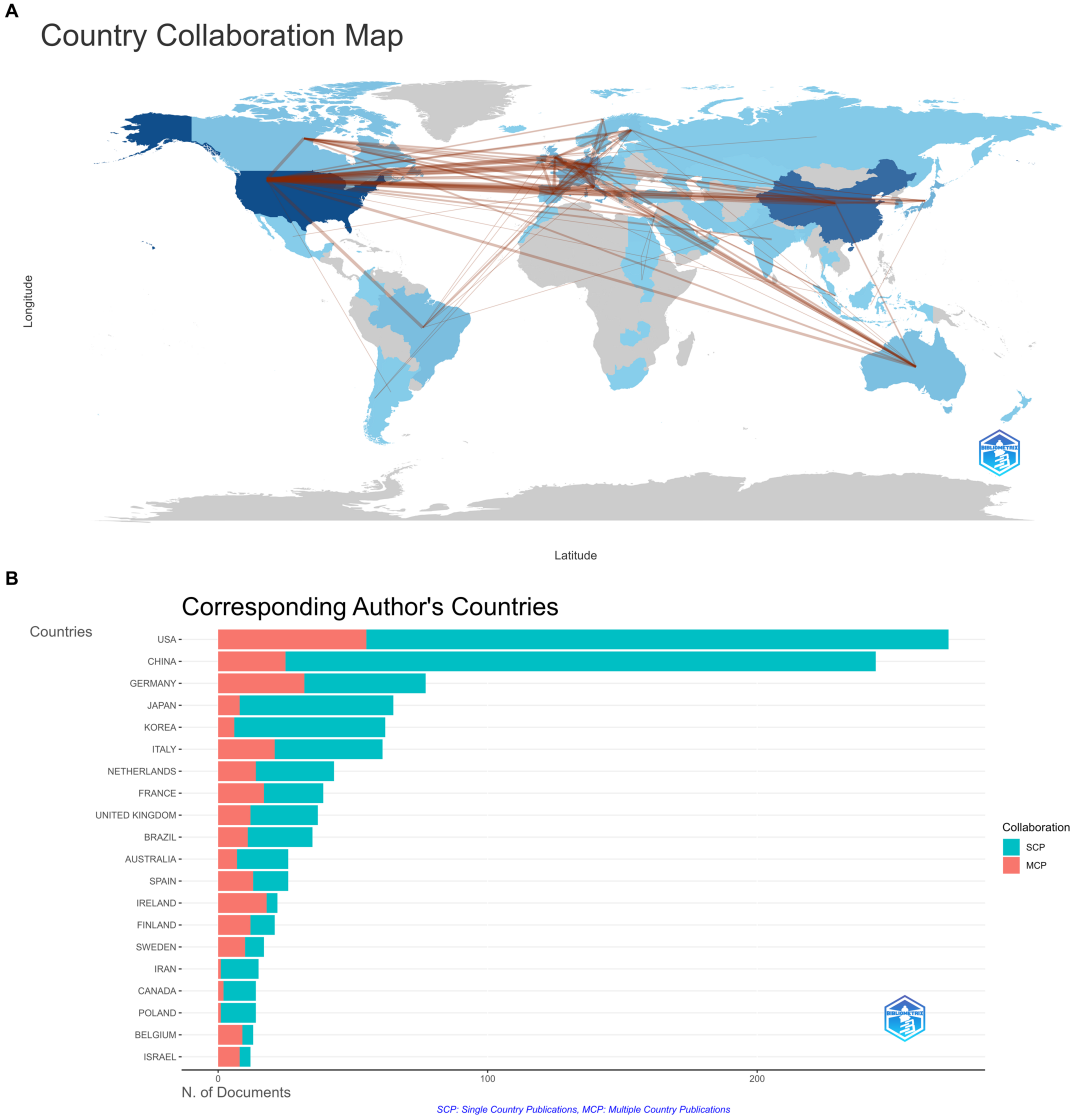
(A) Collaboration world map. The thickness of the line is indicative of the strength of collaboration. (B) Corresponding author’s countries. Single Country Publications (SCP): This refers to independent scientific research conducted by authors from the same country. Multiple Country Publications (MCP): A collaborative effort between authors from two or more countries on the findings of scientific research.

In addition, cooperation (see Figures 4A,B) illustrates that China and the USA have the most cooperation, whereas cooperation among regions is still minimal.

**Figure 5:**
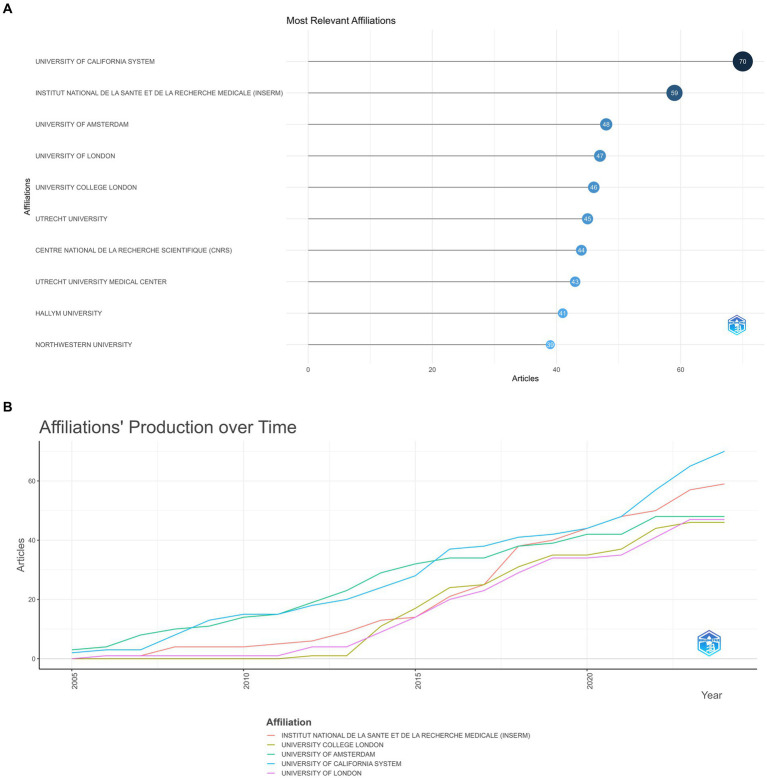
(A) Most relevant affiliation. The size and color of the circle are indicative of the number of publications. (B) Affiliations’ production over time.

Figure 5A indicates the top 10 institutions according to the number of academic outputs. University of California system (70 papers) and Institut National De La Sante Et De La recherche medicale (INSERM) (59 papers) occupied a dominant position, the other eight organizations exhibited little variation in the number of articles they published (Figure 5B).

### 3.3 Sources and co-citation

**Figure 6:**
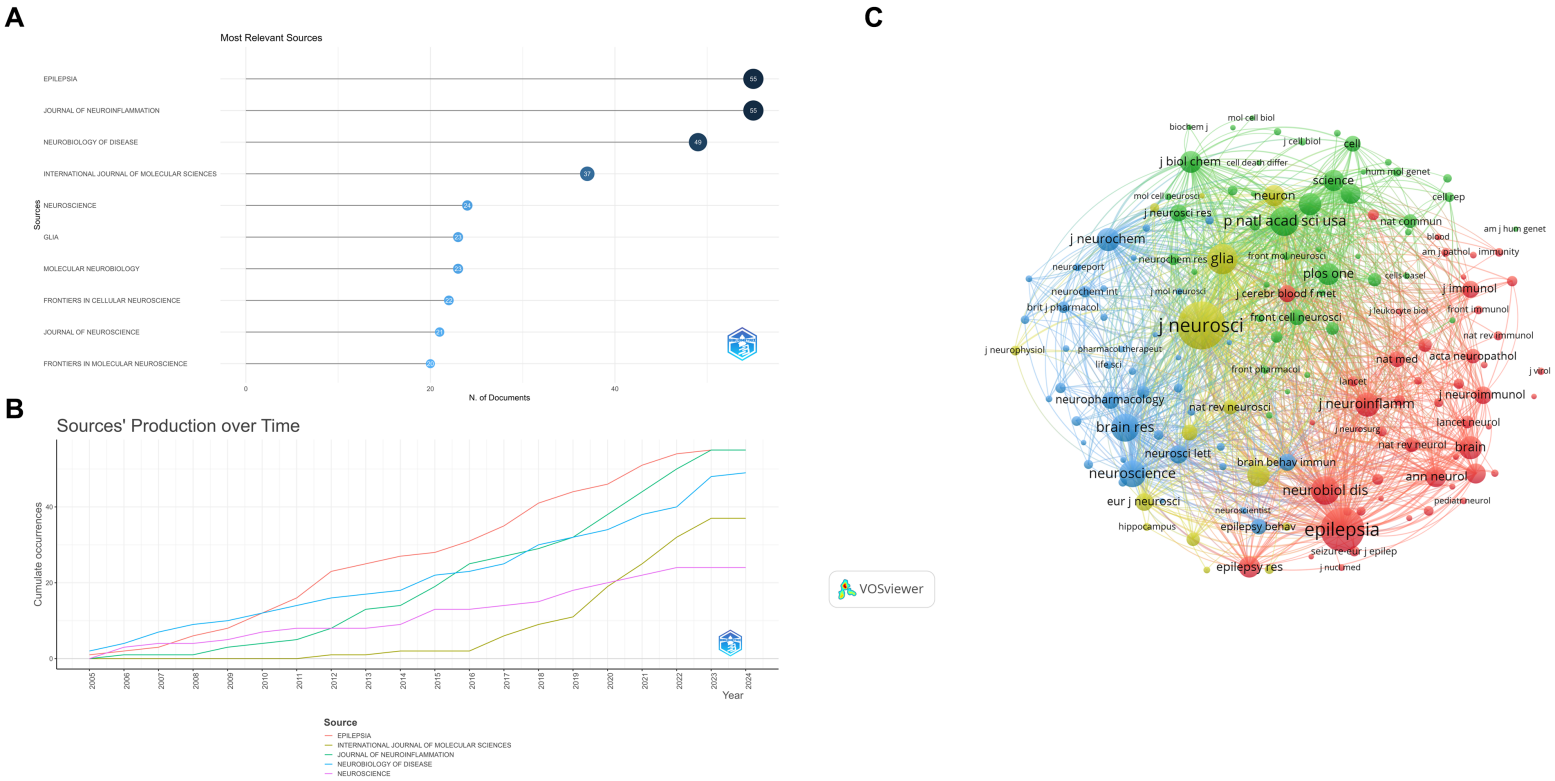
(A) Most relevant sources. The size and color of the circle are indicative of the number of publications. (B) Sources’ production over time. (C) Sources co-citation over 100 times. The color of each circle represents a distinct cluster, the size of the circle represents the frequency of journals, and the thickness of the line represents the strength of association between journals. Yellow cluster: represented by The Journal of Neuroscience. Red cluster: represented by Epilepsia. Green cluster: represented by the National Academy of Sciences. Blue cluster: represented by Brain Research.

This study included 376 sources, of which the top 10 contributed to 329 papers. Epilepsia (*n* = 55, 16.72%) and Journal of Neuroinflammation (*n* = 55, 16.72%) reported the most publications, followed by Neurobiology of Disease (*n* = 49, 14.89%), International Journal of Molecular Sciences (*n* = 37, 11.25%), Neuroscience (*n* = 24, 7.29%), Glia (*n* = 23, 6.99%), Molecular Neurobiology (*n* = 23, 6.99%), Frontiers In Cellular Neuroscience (*n* = 22, 6.69%), Journal of Neuroscience (*n* = 21, 6.38%), and Frontiers in Molecular Neuroscience (*n* = 20, 6.08%; Figures 6A,B).

Journal with more than 100 co-citations analyzed with VOSviewer (Figure 6C). The Journal of Neuroscience (*n* = 4,258) had the largest number of co-citations, followed by Epilepsia (*n* = 3,620), Glia (*n* = 1,918), Proceedings of the National Academy of Sciences (*n* = 1,806), and Neurobiology of Disease (*n* = 1,765).

### 3.4 Authors co-citation

**Figure 7:**
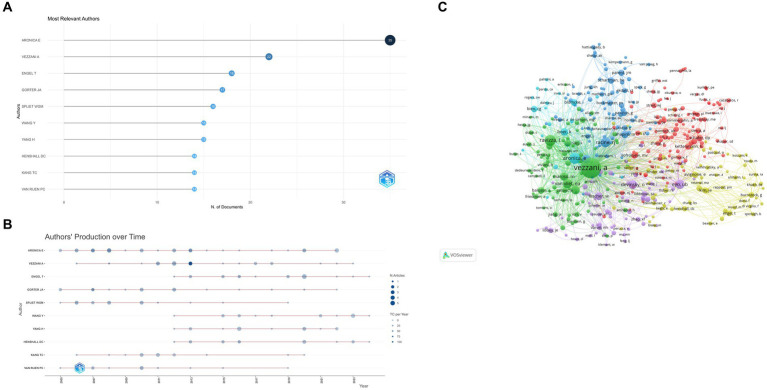
(A) Most relevant authors. The size and color of the circle are indicative of the number of articles published. (B) Authors’ production over time. The size of the circle volume represents the number of publications per year, while the color shade represents the number of citations per year. (C) Authors co-citation over 20 times The color of each circle represents a distinct cluster, the size of the circle represents the frequency of authors, and the thickness of the line represents the strength of association between authors.

Figures 7A,B summarize the 10 most prolific authors and the number of publications over time. The top three are Aronica E (35 papers), Vezzani A (22 papers), and Engel T (18 papers). Strong linkages exist between the clusters, suggesting a high level of cooperation and communication (Figure 7C).

The h-index, g-index, and m-index are metrics used to evaluate the impact and productivity of researchers and their publications. The h-index, proposed by Jorge E Hirsch (10), measures the number of publications that have been cited at least that number of times. The g-index, introduced by Leo Egghe (11), considers the distribution of citations and is defined as the highest rank where the top articles received g^2 citations. The m-index, proposed by Victor A. Mavrodiev, counts the number of papers that have received at least M citations, where M is the maximum number of citations received by any of the researcher’s papers. These indices offer different perspectives on the quality and distribution of citations. Table 1 displays the three aforementioned indexes, as well as TC (total citation), NP (the number of publications), and PY_start (the year for the first publication). This deserves more attention from these authors.

**Table 1:** Author impact.

Element	h_index	g_index	m_index	TC	NP	PY_start
Aronica E	27	35	1.35	3,684	35	2005
Vezzani A	19	22	1	3,155	22	2006
Gorter Ja	15	17	0.75	1,676	17	2005
Spliet Wgm	15	16	0.75	1,427	16	2005
Engel T	14	18	1.077	884	18	2012
Van Rijen Pc	13	14	0.65	1,199	14	2005
Wu Lj	13	13	1.182	1,011	13	2014
Van Vliet Ea	12	14	0.6	1,309	14	2005
Kang Tc	11	14	0.579	520	14	2006
Ravizza T	11	11	0.579	1,482	11	2006

TC, total citation; NP, the number of publications; PY_start: the year for the first publication.

### 3.5 High-cited articles

High citation counts are an indication of an article’s impact and influence within the field. They reflect the article’s ability to stimulate further research, discussion, and application among scholars and practitioners. Table 2 presents the 10 most cited articles from local sources, as defined by the literature searched. The global citation is the total number of citations across all articles.

**Table 2:** Most local cited documents.

Title	Author and Journal	DOI	Year	Local Citations	Global Citations	LC/GC Ratio (%)
Innate and adaptive immunity during epileptogenesis and spontaneous seizures: Evidence from experimental models and human temporal lobe epilepsy	RAVIZZA T, 2008, NEUROBIOL DIS	10.1016/j.nbd.2007.08.012	2008	125	533	23.45
Glia and epilepsy: excitability and inflammation	DEVINSKY O, 2013, TRENDS NEUROSCI	10.1016/j.tins.2012.11.008	2013	119	552	21.56
Blood–brain barrier leakage may lead to progression of temporal lobe epilepsy	VAN VLIET EA, 2007, BRAIN	10.1093/brain/awl318	2007	81	600	13.50
Status Epilepticus Induces a Particular Microglial Activation State Characterized by Enhanced Purinergic Signaling	AVIGNONE E, 2008, J NEUROSCI	10.1523/JNEUROSCI.1820-08.2008	2008	81	217	37.33
Rapid astrocyte and microglial activation following pilocarpine-induced seizures in rats	SHAPIRO LA, 2008, EPILEPSIA	10.1111/j.1528-1167.2008.01491.x	2008	71	179	39.66
Microglia–Neuron Communication in Epilepsy	EYO UB, 2017, GLIA	10.1002/glia.23006	2017	70	172	40.70
Evidence of activated microglia in focal cortical dysplasia	BOER K, 2006, J NEUROIMMUNOL	10.1016/j.jneuroim.2006.01.002	2006	69	120	57.50
Epilepsy and brain inflammation	VEZZANI A, 2013, EXP NEUROL	10.1016/j.expneurol.2011.09.033	2013	69	414	16.67
Neuronal Hyperactivity Recruits Microglial Processes via Neuronal NMDA Receptors and Microglial P2Y12 Receptors after Status Epilepticus	EYO UB, 2014, J NEUROSCI	10.1523/JNEUROSCI.0416-14.2014	2014	68	305	22.30
Complex alterations in microglial M1/M2 markers during the development of epilepsy in two mouse models	BENSON MJ, 2015, EPILEPSIA	10.1111/epi.12960	2015	63	118	53.39

### 3.6 Keywords and theme

#### 3.6.1 Keyword analysis

**Figure 8:**
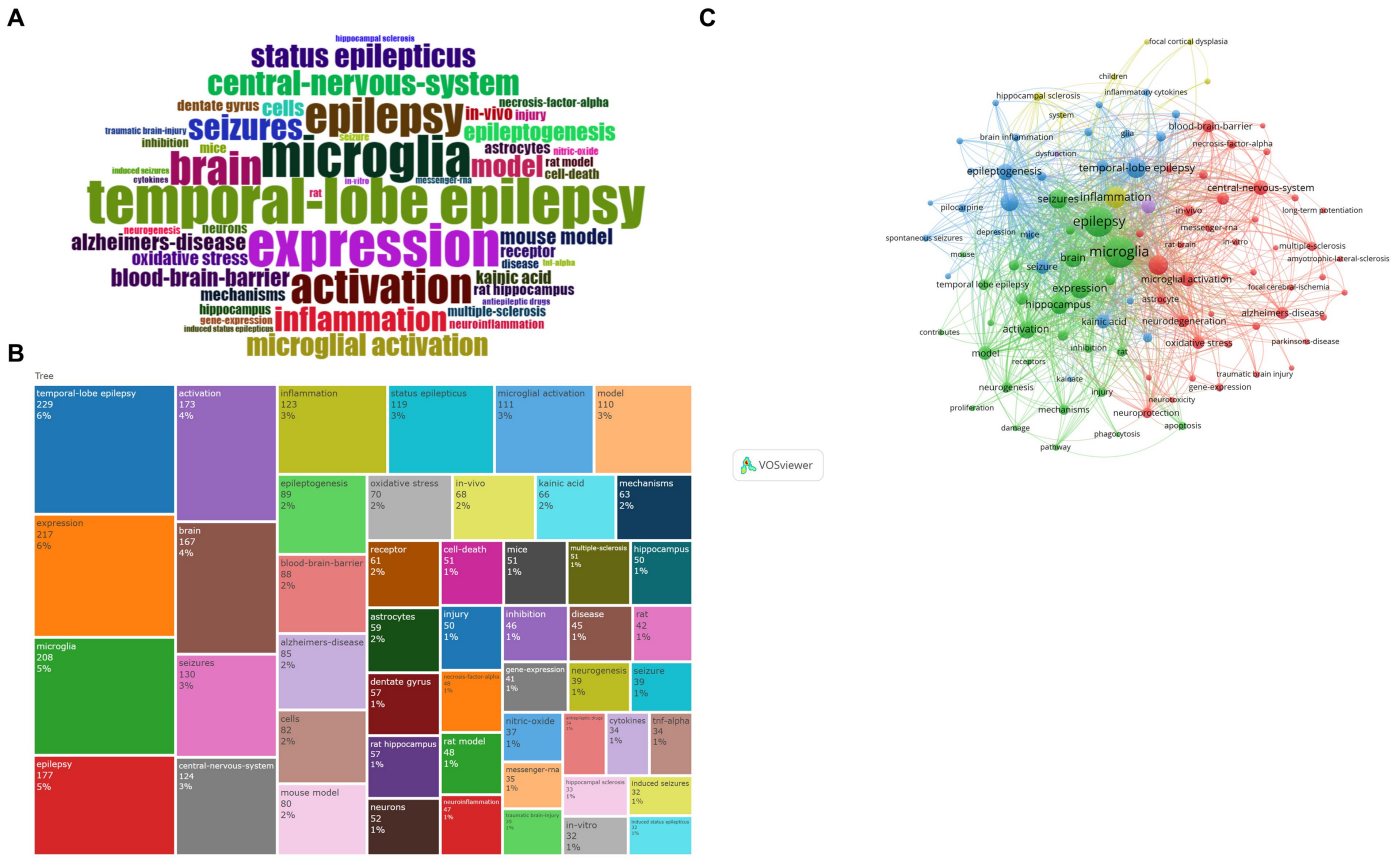
(A) Wordcloud. The font size serves to indicate the frequency of occurrence. (B) Treemap. The area occupied represents the frequency of occurrence. (C) Keyword cluster analysis. The color of each circle represents a distinct cluster, the size of the circle represents the frequency of keywords, and the thickness of the line represents the strength of association between keywords. Green cluster: represented by epilepsy and microglia expression. Blue cluster: represented by temporal lobe epilepsy. Red cluster: represented by microglia activation and other diseases. Yellow cluster: represented by inflammation.

Using the R package Bibliometrix, the most prevalent keywords with frequencies over 150, were TLE (*n* = 229), expression (*n* = 217), microglia (*n* = 208), epilepsy (*n* = 177), activation (*n* = 174), brain (*n* = 166), seizure (*n* = 129), central nervous system (*n* = 124), inflammation (*n* = 123), status epilepticus (*n* = 118), modeling (*n* = 111), and microglia activation (*n* = 109) (Figures 8A,B).

Based on the keywords, about 4 clusters can be identified by VOSviewer: TLE (blue), microglia (green), neuroinflammation (red), and inflammation (yellow) (Figure 8C). This green network primarily exhibits microglia expression and activation during epileptogenesis. This blue network is dedicated to the study of TLE, status epilepticus, and the treatment of epilepsy. The red network primarily depicts microglia activation based on the mouse model for studying neuroinflammation, as well as the link between epilepsy and other diseases, including Alzheimer’s disease and multiple sclerosis. The objective of the yellow network is to concentrate on the general aspects of inflammation.

#### 3.6.2 Thematic evolution and hotspots

**Figure 9:**
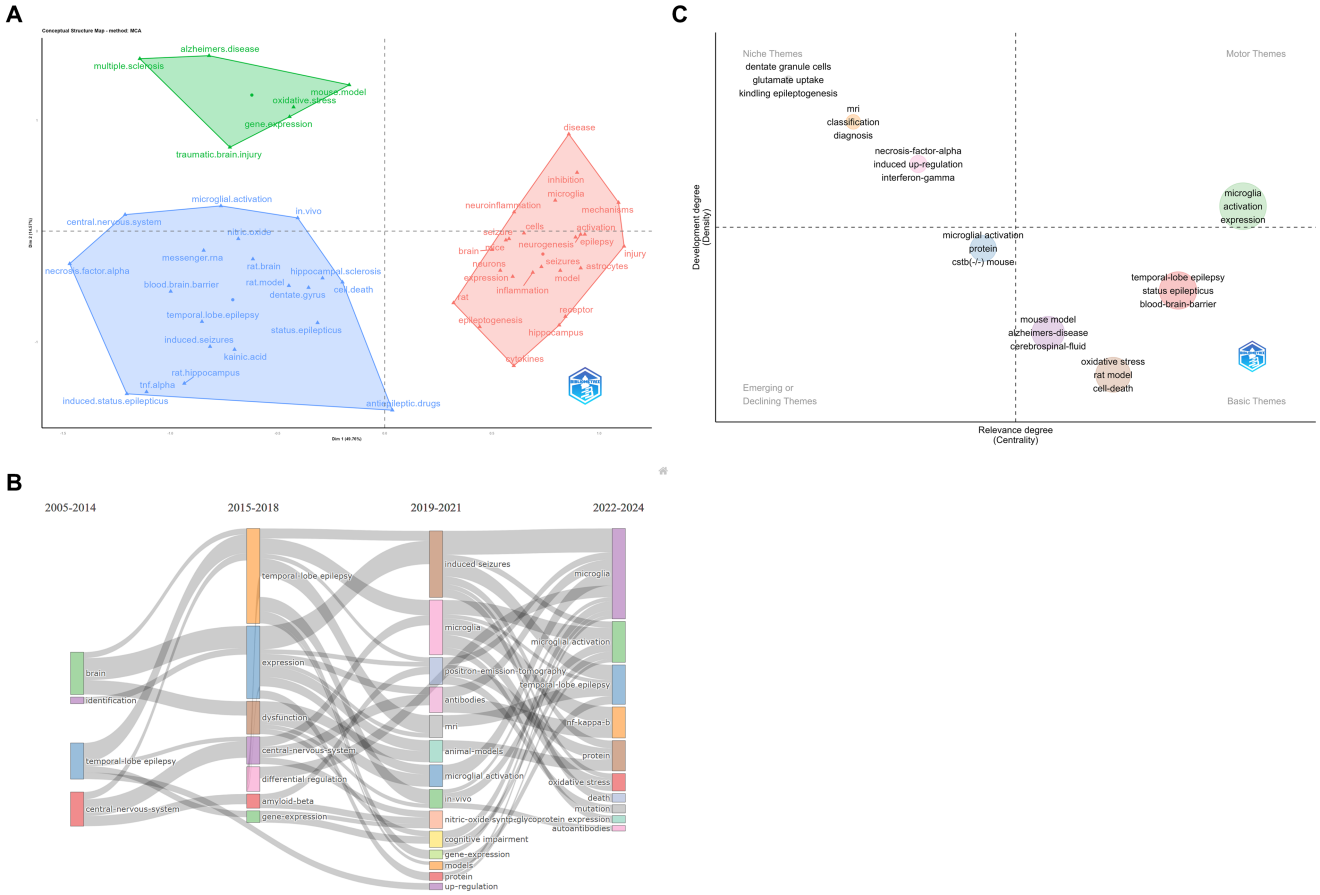
(A) Cluster analysis. (B) Thematic evolution. The thickness of the line represents the strength of association between categories. (C) Thematic map. Quadrant 1: Motor Themes both important and well developed; Quadrant 2: Niche Themes well-developed, but not important to the current field; Quadrant 3: Emerging or Declining Themes fringe themes, also not well developed, probably just emerging, and may soon be superseded; Quadrant 4: Basic Themes important to the field, but not well developed. Typically refers to fundamental concepts. The size of the circle represents the frequency of terms.

The thematic analysis commences with the extraction of pertinent terms from the titles, keywords, and abstracts of related literature. These terms are then subjected to Porter’s stemming algorithm (12), which reduces inflected words to their word stem, base, or root form. This is followed by multiple correspondence analysis (MCA) to apply cluster analysis, which maps conceptual connections (9). Figure 9A reveals three distinct categories that encompass various aspects of epilepsy and its related biological processes.

The first category revolves around TLE, focal epilepsy that originates in the temporal lobe of the brain (13). This cluster highlights specific pathophysiological mechanisms and clinical manifestations associated with this type of epilepsy, including seizure patterns, neurobiological changes, and potential treatment strategies.

The second category focuses on epilepsy-related disorders. This cluster encompasses a wide range of comorbidities and conditions that are often associated with epilepsy, such as Alzheimer’s disease (AD), traumatic brain injury (TBI), and multiple sclerosis (MS).

The third category centers on microglia activation. Microglia are immune cells in the brain that play a crucial role in the immune response and inflammation. In the context of epilepsy, microglia activation has been implicated in seizure generation and epileptogenesis. This cluster explores the role of microglia in epilepsy, including how their activation contributes to seizure activity and potential therapeutic interventions targeting microglia to control seizures.

Sankey diagram (Figure 9B) presents a comprehensive visualization of the evolving research topics and related terminologies spanning from 2005 to 2024. Distinct colors represent different years, and lines connect the years to their respective research terms. The chart reveals a diverse array of topics including ‘brain’, ‘identification’, ‘expression’, and ‘antibodies’, among others. Notably, terms such as ‘microglia’, ‘microglial activation’, ‘microglial expression’, ‘protein’, ‘gene expression’, ‘models’, and ‘neuromodulation’ also play significant roles in the chart.

The chart suggests a shift in research focus over the years. For instance, the initial period (2005–2014) likely focused on fundamental research concerning the brain and its functions, evident in the prevalence of terms like ‘temporal-lobe epilepsy’, ‘induced seizures’, and ‘positron-emission-tomography’. The following years (2015–2018) seem to emphasize more on the role of microglia and its activation in the central nervous system, as indicated by the increased prominence of terms like ‘microglia’, ‘microglial activation’, and ‘nf-kappa-b’.

Subsequently, the chart shows a progression toward understanding the regulation and dysfunction of proteins, genes, and models (2019–2021), reflected in terms like ‘differential regulation’, ‘gene expression’, ‘animal models’, and ‘oxidative stress’. Finally, the chart indicates a trend toward *in-vivo* studies exploring the role of specific molecules like ‘amyloid-beta’ and ‘nitric oxide synthase’ in conditions like ‘cognitive impairment’ and ‘autoantibodies’ (2022–2024).

To study the hotspots in epilepsy research over the past 5 years, we analyze this comprehensive graph (Figure 9C) depicting the evolving relationship between epilepsy and microglia. The graph is segmented into distinct categories, reflecting different levels of importance and development within the field:

Motor Themes: These are the core areas that are both significantly important and well-researched in epilepsy and microglia studies. They represent the current focal points where substantial advancements have been made and continue to shape our understanding of epilepsy’s pathophysiology. Terms associated with these themes, such as “Microglia Activation” and “Expression,” indicate their profound impact on the field’s progression.

Niche Themes: While well-developed, the Niche themes are not considered central to the current epilepsy research field. These include studies on dentate granule cells, glutamate uptake mechanisms, kindling epileptogenesis, MRI techniques, necrosis-factor-alpha, induced up-regulation, and interferon-gamma. These areas provide valuable insights into the pathophysiology of epilepsy but are not the primary focus of recent research.

Emerging or Declining Themes: The Emerging or Declining themes encompass areas that are either newly emerging or potentially fading from the research spotlight. Microglial activation, which overlaps with the Motor themes, is also noted here, indicating its potential for further development or decline depending on future research trends. Additionally, the Cystatin B gene (CSTB) (−) mouse model, a genetic model used to study epilepsy, falls into this category, suggesting a possible shift in research focus. CSTB protein acts as an intracellular thiol protease inhibitor, thereby inhibiting the functionality of tissue proteases. Furthermore, it plays a pivotal role in brain development by regulating various functions in neurons (14). Cstb knockout mice represent a valuable resource for investigating the function of Cstb in the brain. The loss of cerebellar granule cells and Purkinje cells was observed in the cerebellum of Cstb-deficient mice (15). Furthermore, the genetic mouse model of Dravet syndrome (DS) is of considerable interest. In a study conducted by Salazar et al. (16), the activation of microglia was observed in retinal tissue, suggesting that the retina may serve as a potential biomarker for DS.

Basic Themes: The Basic themes represent fundamental concepts in epilepsy research that are important to the field but not yet fully developed. These include mouse and rat models of epilepsy, Alzheimer’s disease (in the context of comorbidity or shared pathophysiological mechanisms), cerebrospinal fluid biomarkers, oxidative stress, cell death mechanisms, TLE, status epilepticus, and the blood–brain barrier. These areas form the foundation for understanding epilepsy but require further investigation to elucidate their precise roles in the disease process.

## 4 Discussion

The current study provides a comprehensive bibliometric analysis of the relationship between microglia and epilepsy, drawing from the extensive literature available in the Web of Science Core Collection (WOSCC). The analysis covers a period spanning from 2005 to 2024, highlighting trends, key contributors, research hotspots, and evolving themes in this field.

The publication trends demonstrate a consistent increase in the number of studies exploring the relationship between microglia and epilepsy over the past two decades. This trend, which is evident in both cumulative and annual publications, highlights the growing interest and recognition of the importance of microglia in epilepsy research. The increase is particularly significant after 2019, indicating that the field has experienced a significant leap in the past few years.

The analysis of countries and affiliations reveals that the United States, China, and Germany are the most productive in this research area. The United States, notably, maintains a strong lead in terms of citations, suggesting that their research outputs are highly impactful. The extensive collaboration among researchers, particularly between China and the United States, suggests the value of international cooperation in tackling complex neuroscientific questions. Such collaborations foster the exchange of ideas, methods, and resources, ultimately leading to more impactful research. The top institutions, such as the University of California system and INSERM, represent hubs of excellence in this research domain.

The analysis of sources and co-citations highlights the journals that are most influential in this field. Epilepsia and Journal of Neuroinflammation emerge as the most productive sources, indicating their importance as platforms for disseminating cutting-edge research on the microglia-epilepsy interface. The large number of co-citations for the Journal of Neuroscience suggests its influence and centrality in the research landscape. The analysis of authors reveals a cluster of highly productive researchers in this area, with Aronica E, Vezzani A, and Engel T leading the way. Their h-index, g-index, and m-index values indicate their scientific impact and productivity. These researchers serve as role models for aspiring scientists in the field.

Finally, the thematic analysis reveals three distinct categories encompassing TLE, epilepsy-related disorders, and microglia activation. The hippocampus, as a representative of the first category, is an inescapable presence in the study of temporal lobe epilepsy and has been demonstrated to exhibit both structural and functional alterations in TLE (4). Some animal models have identified an abnormal enhancement of neurogenesis in the hippocampal dentate gyrus by microglia following epileptogenesis (17, 18). Microglia are also implicated in the dysregulation of excitatory and inhibitory synaptic balance, which is typically excitatory in patients with epilepsy (19). Dysregulation of E/I balance in the hippocampus after epilepsy may lead to epilepsy recurrence (20, 21). When it comes to the cluster of epilepsy-related disorders, one of the most significant contributors to this category is AD, and the key to linking epilepsy, microglia, and AD may be oxidative stress (OS). *In vitro* and *in vivo* studies have indicated that the formation of reactive oxygen species (ROS) and the resulting OS may be involved in the pathogenesis of seizures (22, 23). Microglia are known to express an extensive range of pattern recognition receptors (PRRs) that enable them to detect ROS, subsequently leading to their activation and infiltration at the site of injury (24). Similarly, Aβ proteins have been demonstrated to activate numerous inflammatory pathways in microglia, resulting in the production of pro-inflammatory cytokines and ROS (25). Accumulating evidence suggests that the release of neurotransmitters, such as glutamate and gamma-aminobutyric acid (GABA) (26), as well as inflammatory cytokines and chemokines, contribute to microglia activation and neuroinflammation. For example, Zhang et al. (27) demonstrated that increased glutamate levels in the epileptic brain activate microglia and promote the release of inflammatory cytokines, which in turn exacerbate neuronal excitability and epileptogenesis. Cytokines, such as interleukin-1β (IL-1β) and tumor necrosis factor-*α* (TNF-α), have been shown to increase neuronal excitability by modulating voltage-gated ion channels and excitatory synaptic transmission (28–30). In addition, microglia-derived reactive oxygen species can also contribute to epileptogenesis by disrupting the delicate balance between excitation and inhibition in the CNS (31–33).

The evolving research topics over the years, reflected in the Sankey diagram, indicate a shift from fundamental research on brain functions to a deeper understanding of the regulation and dysfunction of proteins, genes, and models. Recent research hotspots focus on *in-vivo* studies exploring the role of specific molecules in epilepsy and its comorbidities. In addition to the previously discussed pro-inflammatory molecules, the nuclear factor-κB (NF-κB) pathway is also of significance in recent years. NF-κB activation is mediated by two major signaling pathways, namely the canonical and the non-canonical NF-κB signaling pathways (34). There has been controversy about the role of NF-κB in seizure neurons, which may be attributed to the activation of different NF-κB subunits (35). However, for microglia, some studies showed the toll-like receptor 4 (TLR4) /NF-κB signaling pathway may be implicated in the activation and polarization of epileptic microglia that results in increased brain damage (36, 37). Similarly, as a principal constituent of the canonical pathway, TGF-*β* activated kinase 1 (TAK1) is activated in microglia following experimental induction of epilepsy and contributes to the pathogenesis of epilepsy (38). Therefore, NF-κB signaling pathway is worthy of further investigation.

Based on the current analysis, several promising directions for future research emerge. Firstly, there is a need for further exploration of the specific molecular mechanisms underlying microglia activation in epilepsy. Secondly, more studies utilizing animal models and *in-vivo* approaches could provide critical insights into the role of microglia in epileptogenesis. Finally, interdisciplinary approaches combining neuroscience, immunology, and other disciplines could help unlock new avenues for therapeutic interventions targeting microglia in epilepsy.

## 5 Conclusion

In conclusion, this bibliometric study reveals a surge in epilepsy-microglia research, led by key countries, journals, and researchers. Temporal lobe epilepsy, epilepsy-related disorders, and microglia activation are focal themes. Future directions include exploring microglia activation mechanisms, utilizing animal models, and interdisciplinary approaches.

## Data Availability

The raw data supporting the conclusions of this article will be made available by the authors, without undue reservation.
